# Metoprolol or Verapamil in the Management of Patients With Hypertrophic Cardiomyopathy: A Systematic Review

**DOI:** 10.7759/cureus.43197

**Published:** 2023-08-09

**Authors:** Maher Taha, Purva Dahat, Stacy Toriola, Travis Satnarine, Zareen Zohara, Ademiniyi Adelekun, Kofi D Seffah, Korlos Salib, Lana Dardari, Ana P Arcia Franchini

**Affiliations:** 1 Internal Medicine, California Institute of Behavioral Neurosciences and Psychology, Fairfield, USA; 2 Pathology, California Institute of Behavioral Neurosciences and Psychology, Fairfield, USA; 3 Pediatrics, California Institute of Behavioral Neurosciences and Psychology, Fairfield, USA; 4 Family Medicine, California Institute of Behavioral Neurosciences and Psychology, Fairfield, USA; 5 Research, California Institute of Behavioral Neurosciences and Psychology, Fairfield, USA

**Keywords:** beta-blockers, left ventricular outflow obstruction, sudden cardiac death (scd), calcium channel blockers, verapamil, metoprolol, hocm

## Abstract

Hypertrophic cardiomyopathy (HCM) is the most common genetic heart disease and is a prevalent cause of sudden cardiac death (SCD). This study aims to establish the benefits and therapeutic value metoprolol or verapamil offer to patients who suffer from symptoms caused by HCM, with regard to resolving left ventricular outflow tract obstruction (LVOTO), as well as improving a patient’s quality of life and reducing symptoms. We conducted a systematic review to find clinical studies that described the use of metoprolol or verapamil in the management of HCM. Three databases were analyzed for studies, PubMed, Google Scholar, and ScienceDirect. We discovered 6,260 potentially eligible records across all the databases. According to our eligibility criteria, we included four studies in this review. Metoprolol showed median left ventricular outflow tract (LVOT) gradients of 25 mm Hg versus 72 mm Hg (P = 0.007) at rest, 28 mm Hg versus 62 mm Hg (P < 0.001) at peak exercise, and 45 mm Hg versus 115 mm Hg (P < 0.001) post-exercise. Verapamil also showed a statistically significant increase in exercise capacity. Both drugs have been shown to be safe to use with a good side effect profile; however, metoprolol was better tolerated in the patient population that was tested in the studies collected. In this study, metoprolol was effective in reducing LVOT and improving the quality of life in patients, while verapamil showed variable effects on both exercise capacity and baseline hemodynamics.

## Introduction and background

Hypertrophic cardiomyopathy (HCM) is a relatively common and complex genetic disease and is thought to affect as many as one in 500 people [1,2]. HCM affects patients of both genders and several ethnic groups and has become widely recognized as an important cause of sudden cardiac death (SCD). It is characterized by left ventricular hypertrophy unexplained by secondary causes and a non-dilated left ventricle (LV) with a preserved or increased ejection fraction. It is commonly asymmetric, with the most severe hypertrophy involving the basal interventricular septum [3]. Familial HCM is inherited in an autosomal dominant (AD) fashion, implying that one duplicate of the changed gene is sufficient to cause the problem. The two duplicates can be affected, prompting more extreme signs and side effects of HCM. Non-familial hypertrophic cardiomyopathy is not inherited [4]. Multiple gene mutations (also known as variants) can cause familial HCM; the most commonly involved genes are *MYH7*, *MYBPC3*, *TNNT2*, and *TNNI3*. The protein delivered from the *MYH7* gene, called cardiac β-myosin heavy chain, is the primary part of the thick filament in sarcomeres, a central recurrent unit inside the muscle that controls contraction. The protein derived from the *MYBPC3* gene, cardiac myosin-restricting protein C, partners with the thick filament, which is responsible for providing structural support and assisting with regulating muscle contractions [5]. Both *TNNT2* and *TNNI3* genes are responsible for the instructions that lead to the production of cardiac troponin T and cardiac troponin I, respectively, two of the three proteins that make up the troponin protein complex tracked down in cardiac muscle cells. The troponin complex is associated with the thin filament of sarcomeres and controls muscle contraction and relaxation by regulating the interaction of filaments [4].

Clinical manifestations of HCM

According to the American Heart Association, HCM can present with many signs and symptoms, including chest pain and shortness of breath, exacerbated with physical exertion, fatigue, arrhythmias, dizziness, lightheadedness, syncope, and edema in the ankles, feet, legs, and abdomen [6]. HCM is a clinically progressive disease, meaning that as the disease course progresses, it becomes more severe and can lead to a poorer quality of life and severe complications, making it a critical disease to recognize early and diagnose correctly.

Diagnosis of HCM

Patients referred with a clinical suspicion of HCM should undergo electrocardiogram (ECG) monitoring, a chest X-ray (CXR), and a transthoracic echocardiogram (TTE) Doppler examination. An ECG in HCM may be normal with mild degrees of hypertrophy or show left ventricular hypertrophy. Abnormal Q waves, which may mimic myocardial infarction and reflect septal hypertrophy, are a feature of HCM. Other ECG features of HCM are sharply negative T waves, particularly in precordial leads V3-V5 [7,8]. A chest X-ray (CXR) can either indicate normal results or reveal enlargement of the LV or left or right atrium, or both. This may or may not be accompanied by vascular redistribution in the lungs. The aorta is usually small in size. If there is a protrusion on the left border of the heart, located between the left atrial appendage and the left ventricular apex, it could suggest an extension of anteroseptal hypertrophy into the anterolateral wall [7]. The most critical laboratory investigation to diagnose HCM is the TTE Doppler examination. This can identify various aspects of the condition, such as the location and extent of hypertrophy, systolic and diastolic function, the presence and severity of systolic anterior motion, the degree of subaortic and/or midventricular obstruction, the direction and degree of mitral regurgitation, the size of the left atrium, and the presence of additional mitral valve abnormalities [7]. Mitral regurgitation caused by the systolic anterior motion of the anterior mitral leaflet is directed toward the left atrium posteriorly. If the mitral regurgitation is directed anteriorly or centrally, it may indicate additional mitral valve abnormalities such as abnormal papillary muscles or prolapse. TTE Doppler studies are particularly beneficial in identifying these additional mitral valve abnormalities and distinguishing the type of obstruction present in the LV. Patients who do not display any outflow obstruction at rest should undergo appropriate provocation to determine if there is evidence of latent obstruction using echocardiography/Doppler [7,9,10].

Left ventricular outflow tract (LVOT) and left ventricular ejection fraction (LVEF)

LVOT gradient is often used to evaluate the severity of the disease, the presence or absence of LVOT obstruction, and the efficacy of treatment [11]. In HCM, the LVOT can be obstructed (LVOTO), which is caused by contact between the anterior leaflet of the mitral valve and the interventricular septum during systole. LVOTO has been defined as a peak instantaneous gradient at left ventricular outflow of at least 30 mm Hg, either at rest or provocation [12]. Approximately one-quarter of patients with HCM have LVOTO at rest; however, some patients without outflow obstruction at rest can have their gradients altered by physiological and pharmacological interventions [12,13]. This leads to a diminished LV end-diastolic volume or increased LV contractility (latent LVOTO). LVOTO causes an acute decrease in cardiac output, increased LV filling pressures, and myocardial ischemia, which can result in symptoms associated with HCM, such as chest pain, exertional dyspnea, presyncope, and syncope.

LVEF, an index of LV contractility, indicates the degree of change in LV volume from diastole to systole. LVEF can be calculated using the LV end-diastolic volume, subtracting the LV end-systolic volume from it, and dividing it by the end-diastolic volume [13]. LV contractility can remain normal in HCM, which may result in a normal LVEF; as the disease progresses, the left ventricle has a small volume and needs to be emptied to maintain cardiac output; therefore, LVEF can be elevated in HCM [14].

Pharmacological management with metoprolol and verapamil

Due to the factors described above, especially LVOTO, which causes many of the symptoms seen in HCM, the symptoms are often managed with beta-blockers and calcium channel blockers (CCBs), the two of which are the focus of this review. Metoprolol is a cardioselective beta-1-adrenergic receptor inhibitor that competitively blocks beta-1-receptors; metoprolol does not exhibit membrane-stabilizing or intrinsic sympathomimetic activity [15]. For a long time, metoprolol has been considered the first-line treatment for managing symptomatic HCM. It is thought to be based on its mechanism of action (MOA), which is that the decrease in heart rate (HR), and contractility allows for more time during diastolic filling, as well as decreasing the LVOT gradient. This would then lead to improved symptoms and an ability to remain asymptomatic and an improved exercise capacity. Another drug widely used in the treatment of HCM is verapamil, a non-dihydropyridine calcium channel blocker (CCB). CCBs inhibit the entry of calcium ions into the slow L-type calcium channels in the myocardium and vascular smooth muscle during depolarization. This inhibition will produce coronary vascular smooth muscle relaxation and coronary vasodilation. Verapamil also increases myocardial oxygen delivery. Verapamil correlates with adverse chronotropic effects and decreased sympathetic nervous system activity [16]. Both drugs are widely available and used in the treatment of patients with HCM; therefore, it is essential to know why they are so commonly used, the effect they have on patients with HCM, and how they improve patients’ lives and, if possible, to determine which of the two is more effective in the management of HCM.

## Review

Methods

We implemented the Preferred Reporting Items for Systematic Reviews and Meta-Analyses (PRISMA) 2020 guidelines to design and describe the findings of this systematic literature review [17].

Search Strategy

We carried out an extensive search in PubMed, Google Scholar, and ScienceDirect. We used appropriate keywords and Medical Subject Headings (MeSH) terms to identify all potentially relevant articles that include the use of metoprolol or verapamil in the treatment of HCM and its effect on cardiac physiology and left ventricular outflow tract obstruction. We applied the Boolean method to combine the keywords and MeSH terms to synthesize a uniform search through the various databases; the results are shown in Table 1. We also used keywords certain to our topic to search Google Scholar and ScienceDirect databases; the results are shown in Table 2.

**Table 1:** Results of the MeSH strategy used to identify concepts and search for studies in the PubMed database MeSH: Medical Subject Headings

MeSH concept	Database	Number of studies (with filters)
Metoprolol OR Beta Blocker OR Anti-Hypertensive OR Vasodilator OR ("Metoprolol/administration and dosage"[Majr] OR "Metoprolol/adverse effects"[Majr] OR "Metoprolol/agonists"[Majr] OR "Metoprolol/analogs and derivatives"[Majr] OR "Metoprolol/antagonists and inhibitors"[Majr] OR "Metoprolol/blood"[Majr] OR "Metoprolol/classification"[Majr] OR "Metoprolol/metabolism"[Majr] OR "Metoprolol/pharmacokinetics"[Majr] OR "Metoprolol/pharmacology"[Majr] OR "Metoprolol/therapeutic use"[Majr] OR "Metoprolol/toxicity"[Majr])	PubMed	55,367
Verapamil OR Calcium Channel Blocker OR Nondihydropyridine Calcium Channel Blockers OR ("Verapamil/administration and dosage"[Majr] OR "Verapamil/adverse effects"[Majr] OR "Verapamil/agonists"[Majr] OR "Verapamil/analogs and derivatives"[Majr] OR "Verapamil/antagonists and inhibitors"[Majr] OR "Verapamil/blood"[Majr] OR "Verapamil/classification"[Majr] OR "Verapamil/metabolism"[Majr] OR "Verapamil/pharmacokinetics"[Majr] OR "Verapamil/pharmacology"[Majr] OR "Verapamil/therapeutic use"[Majr] OR "Verapamil/toxicity"[Majr])	PubMed	12,736
Hypertrophic Cardiomyopathy or Cardiomegaly or Sudden Cardiac Death OR ("Cardiomyopathy, Hypertrophic/classification"[Majr] OR "Cardiomyopathy, Hypertrophic/complications"[Majr] OR "Cardiomyopathy, Hypertrophic/diagnosis"[Majr] OR "Cardiomyopathy, Hypertrophic/diet therapy"[Majr] OR "Cardiomyopathy, Hypertrophic/drug therapy"[Majr] OR "Cardiomyopathy, Hypertrophic/epidemiology"[Majr] OR "Cardiomyopathy, Hypertrophic/etiology"[Majr] OR "Cardiomyopathy, Hypertrophic/genetics"[Majr] OR "Cardiomyopathy, Hypertrophic/mortality"[Majr] OR "Cardiomyopathy, Hypertrophic/physiopathology"[Majr] OR "Cardiomyopathy, Hypertrophic/prevention and control"[Majr] OR "Cardiomyopathy, Hypertrophic/rehabilitation"[Majr] OR "Cardiomyopathy, Hypertrophic/therapy"[Majr])	PubMed	4,782
Left Ventricular Outflow Obstruction OR Cardiac Stenosis OR Septal Hypertrophy OR ("Ventricular Outflow Obstruction, Left/classification"[Mesh] OR "Ventricular Outflow Obstruction, Left/complications"[Mesh] OR "Ventricular Outflow Obstruction, Left/diagnosis"[Mesh] OR "Ventricular Outflow Obstruction, Left/drug therapy"[Mesh] OR "Ventricular Outflow Obstruction, Left/epidemiology"[Mesh] OR "Ventricular Outflow Obstruction, Left/etiology"[Mesh] OR "Ventricular Outflow Obstruction, Left/pathology"[Mesh] OR "Ventricular Outflow Obstruction, Left/physiopathology"[Mesh] OR "Ventricular Outflow Obstruction, Left/prevention and control"[Mesh] OR "Ventricular Outflow Obstruction, Left/therapy"[Mesh])	PubMed	4,847
Metoprolol OR Beta Blocker OR Anti-Hypertensive OR Vasodilator OR ("Metoprolol/administration and dosage"[Majr] OR "Metoprolol/adverse effects"[Majr] OR "Metoprolol/agonists"[Majr] OR "Metoprolol/analogs and derivatives"[Majr] OR "Metoprolol/antagonists and inhibitors"[Majr] OR "Metoprolol/blood"[Majr] OR "Metoprolol/classification"[Majr] OR "Metoprolol/metabolism"[Majr] OR "Metoprolol/pharmacokinetics"[Majr] OR "Metoprolol/pharmacology"[Majr] OR "Metoprolol/therapeutic use"[Majr] OR "Metoprolol/toxicity"[Majr]) AND Verapamil OR Calcium Channel Blocker OR Nondihydropyridine Calcium Channel Blockers OR ("Verapamil/administration and dosage"[Majr] OR "Verapamil/adverse effects"[Majr] OR "Verapamil/agonists"[Majr] OR "Verapamil/analogs and derivatives"[Majr] OR "Verapamil/antagonists and inhibitors"[Majr] OR "Verapamil/blood"[Majr] OR "Verapamil/classification"[Majr] OR "Verapamil/metabolism"[Majr] OR "Verapamil/pharmacokinetics"[Majr] OR "Verapamil/pharmacology"[Majr] OR "Verapamil/therapeutic use"[Majr] OR "Verapamil/toxicity"[Majr]) AND Hypertrophic Cardiomyopathy or Cardiomegaly or Sudden Cardiac Death OR ("Cardiomyopathy, Hypertrophic/classification"[Majr] OR "Cardiomyopathy, Hypertrophic/complications"[Majr] OR "Cardiomyopathy, Hypertrophic/diagnosis"[Majr] OR "Cardiomyopathy, Hypertrophic/diet therapy"[Majr] OR "Cardiomyopathy, Hypertrophic/drug therapy"[Majr] OR "Cardiomyopathy, Hypertrophic/epidemiology"[Majr] OR "Cardiomyopathy, Hypertrophic/etiology"[Majr] OR "Cardiomyopathy, Hypertrophic/genetics"[Majr] OR "Cardiomyopathy, Hypertrophic/mortality"[Majr] OR "Cardiomyopathy, Hypertrophic/physiopathology"[Majr] OR "Cardiomyopathy, Hypertrophic/prevention and control"[Majr] OR "Cardiomyopathy, Hypertrophic/rehabilitation"[Majr] OR "Cardiomyopathy, Hypertrophic/therapy"[Majr]) AND Left Ventricular Outflow Obstruction OR Cardiac Stenosis OR Septal Hypertrophy OR ("Ventricular Outflow Obstruction, Left/classification"[Mesh] OR "Ventricular Outflow Obstruction, Left/complications"[Mesh] OR "Ventricular Outflow Obstruction, Left/diagnosis"[Mesh] OR "Ventricular Outflow Obstruction, Left/drug therapy"[Mesh] OR "Ventricular Outflow Obstruction, Left/epidemiology"[Mesh] OR "Ventricular Outflow Obstruction, Left/etiology"[Mesh] OR "Ventricular Outflow Obstruction, Left/pathology"[Mesh] OR "Ventricular Outflow Obstruction, Left/physiopathology"[Mesh] OR "Ventricular Outflow Obstruction, Left/prevention and control"[Mesh] OR "Ventricular Outflow Obstruction, Left/therapy"[Mesh])	PubMed	4,804

**Table 2:** Results of using identified concepts to search for studies in Google Scholar and ScienceDirect databases

Key terms	Database (filters)	Number of studies
Metoprolol and hypertrophic cardiomyopathy	Google Scholar	13,900
	ScienceDirect	194
Verapamil and hypertrophic cardiomyopathy	Google Scholar	11,200
	ScienceDirect	362
Metoprolol and left ventricular outflow obstruction	Google Scholar	12,600
	ScienceDirect	86
Verapamil and left ventricular outflow obstruction	Google Scholar	12,000
	ScienceDirect	177

Inclusion and Exclusion Criteria

We restricted our search to online records available as free full texts, including human participants only. There were no gender or ethnic restrictions. Studies that were selected for inclusion include a clinical diagnosis of HCM. We restricted our choice of studies to clinical trials, randomized controlled trials, systematic reviews, and meta-analyses.

Data Selection and Extraction

Two researchers (MT and PD) independently selected and extracted the relevant studies. The two researchers resolved any issues regarding eligibility by discussing the study design, intervention implemented, outcomes measured, and most importantly, the relevance to our inclusion and exclusion criteria. At times when we could not agree on eligibility, a third reviewer (ST) would help resolve the issue.

We identified a total of 6,260 potentially eligible records across all the databases. Based on our criteria, four reports were established, three of which were randomized crossover trials and a systematic review and meta-analysis. From the studies included, we retrieved the following data: (a) the surname of the principal author and the publication date, (b) the study overview, and (c) the general characteristics of the study population (i.e., mean age, intervention, New York Heart Association (NYHA) class (where applicable), dose and frequency of intervention, and hemodynamic findings, e.g., heart rate, blood pressure, and left ventricular outflow tract gradient).

Study Quality Appraisal

We assessed each study for the potential risk of bias (RoB). We evaluated the crossover trials using the revised Cochrane Risk of Bias 2 (RoB 2) tool [18], which was specialized for crossover trials. Using the Cochrane RoB 2 tool, each randomized crossover trial was evaluated for potential biases based on six domains. Each risk of bias was scored as either low risk, high risk, or some concern for potential bias, depending on the answers to the questions each domain posed. Subsequently, the overall risk of bias was also reported as evoking low, high, or some concern for possible bias. Table 3 demonstrates the results of the revised Cochrane RoB 2 tool for crossover trials.

**Table 3:** Assessment of crossover trials using the Cochrane RoB tool RoB: Risk of Bias, LR: low risk, SC: some concern, HR: high risk

First author (year)	Randomization process	Bias of carryover effects	Deviations from intended interventions	Missing outcome data	Measurement of outcome	Data selection	Overall bias
Dybro et al. (2021) [19]	LR	LR	LR	LR	LR	LR	LR
Gilligan et al. (1993) [20]	LR	LR	LR	LR	LR	LR	LR
Toshima et al. (1996) [21]	LR	LR	LR	LR	LR	LR	LR

We used the Assessment of Multiple Systematic Reviews 2 (AMSTAR 2) tool [22], a quality appraisal tool based on 16 questions, to assess the systematic review and meta-analyses. We appraised the study quality as critically low, low, moderate, or high. Table 4 shows the results of the AMSTAR 2 tool.

**Table 4:** Summary of the AMSTAR 2 tool AMSTAR 2: Assessment of Multiple Systematic Reviews 2, RoB: Risk of Bias, Y: yes, PY: probably yes, N: no, NA: not applicable, PICO: Population, Intervention, Control, and Outcomes

First author (year)	PICO framework included	Pre-defined methods and research proposal	Study design included	Comprehensive literature search strategy	Study selection in duplicate	Data extraction in duplicate	List of excluded studies and justify the exclusions	Detailed description of the included studies	Adequate RoB procedure followed	Disclosure of funding sources	Appropriate statistical analysis	Assess the potential impact of RoB in individual studies on the results of the meta-analysis or other evidence synthesis	Account for RoB in individual studies	Investigation of heterogeneity	Small study bias	Potential conflicts reported	Final quality appraisal of the review
Bayonas-Ruiz et al. (2022) [23]	Y	Y	Y	Y	Y	Y	Y	Y	N	Y	Y	N	N	N	N	Y	High

Results

**Figure 1:**
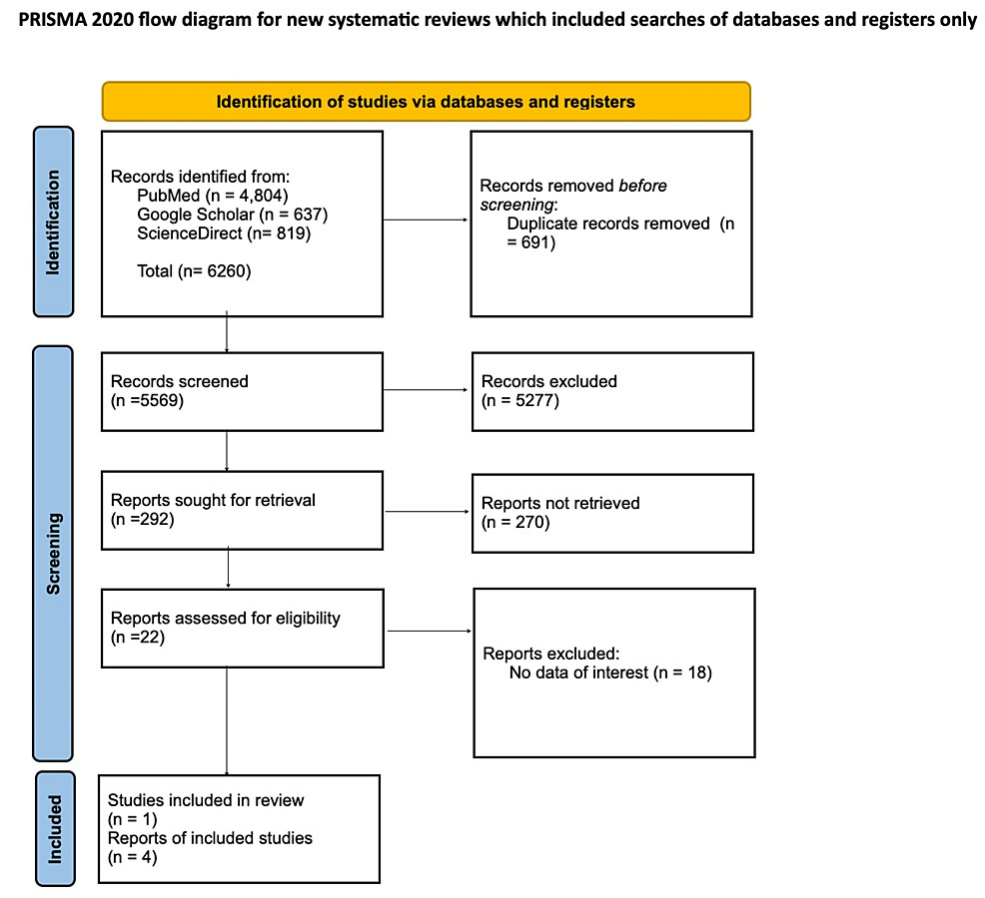
PRISMA flowchart PRISMA: Preferred Reporting Items for Systematic Reviews and Meta-Analyses

We identified a total of 6,260 potentially eligible articles for this review. A total of 4,804 originated from PubMed, 637 from Google Scholar, and 819 from ScienceDirect. No other resources were used. Endnote Online was used to remove any duplicate articles found from each database; this resulted in the removal of 691 duplicate articles before the screening process. The remaining 5,569 articles were thoroughly screened for relevance based on titles and abstracts, after which 5,277 articles were excluded due to their irrelevance to the topic, research objectives, and inclusion and exclusion criteria, and after a second round of screening, 270 more additional articles were removed from eligibility, leaving 22 articles with potential value for this review. These 22 articles were then extensively assessed for their data, and 18 were found to have no data of interest that was of value to the review and were removed, leaving four articles to be included. These included three randomized crossover trials and one systematic review (which was also a meta-analysis). Our review's complete PRISMA flow diagram is shown in Figure 1, and Table 5 and Table 6 summarize the studies selected regarding the baseline characteristics of patients, interventions, and outcomes.

**Table 5:** Details of baseline characteristics of patients involved in the studies selected for this review Values are mean ± SD or median (IQR). SD: standard deviation, IQR: interquartile range, SR: systematic review, MA: meta-analysis, NRT: non-randomized trial, NHYA: New York Heart Association, N/A: not available, LVOT: left ventricular outflow tract, DB: double blind, P-C: placebo-controlled, CT: crossover trial, HR: heart rate, BP: blood pressure, BPM: beats per minute, FC: functional class, E/A ratio: ratio of peak early (E) to peak late (A) transmitral flow velocities, IVS: interventricular septum, Seq A: diltiazem first and then verapamil, Seq B: verapamil first and then diltiazem, n: number of people, PoE: post-exercise, SBP: systolic blood pressure, DBP: diastolic blood pressure

First author (year)	Study design	Sample size	Mean age (years)	Baseline hemodynamics	Baseline NYHA class (n)	Baseline IVS (mm) and LVEF (%)	Baseline LVOT gradient (mm Hg)
Dybro et al. (2021) [19]	DB, P-C, CT	29	60 ± 11	HR (BPM): 79 ± 16, SBP (mm Hg): 121 ± 18, DBP (mm Hg): 82 ± 13	FC 2: 18, FC 3: 11	IVS: 19.8 ± 5, LVEF: 71 ± 8	Rest: 74 (21-97), Valsalva: 117 (66-146), PoE: 178 (135-220)
Gilligan et al. (1993) [20]	DB, P-C, CT	18	37 ± 19	N/A	FC 1: 8, FC 2: 10	IVS: 19 ± 4, LVEF: N/A	At rest: 23 ± 27
Toshima et al. (1986) [21]	DB, P-C, CT	32	42 ± 15	Seq A: HR: 64 ± 7, SBP: 115 ± 13, DBP: 60 ± 12; Seq B: HR: 65 ± 10, SBP: 123 ± 10, DBP: 74 ± 10	Seq A: FC 1: 2, FC 2: 12, FC 3: 0; Seq B: FC 1: 5, FC 2: 9, FC 3: 4	IVS: N/A, LVEF: Seq A: 79 ± 15; Seq B: 83 ± 11	N/A
Bayonas-Ruiz et al. (2022) [23]	SR + MA, data extracted from Bonow et al. (1985) and Lösse et al. (1983)	V: 80	Bonow et al.: 47, Lösse et al.: 45 ± 3	N/A	Lösse et al.: FC: 2.8 ± 0.6	Bonow et al.: LVEF: 71 ± 9	Lösse et al.: rest: 36 ± 7, peak: 100 ± 12

**Table 6:** Details summarizing the effect of each intervention from the studies selected in this review Values are mean ± SD or median (IQR). SD: standard deviation, IQR: interquartile range, P: placebo, WO: washout period, M: metoprolol, V: verapamil, N: nadolol, PoE: post-exercise, PeE: peak-exercise, CO: crossover, OD: once daily, BD: twice daily, n: number of people, mg: milligrams, kg: kilograms, min: minute, FC: functional class, Seq A: diltiazem first and then verapamil, Seq B: verapamil first and then diltiazem

First author (year)	Interventions	Dose of intervention	Hemodynamics after intervention		NYHA class after intervention (n)	LVEF (%) after intervention	LVOT gradient (mm Hg) after intervention
Dybro et al. (2021) [19]	P-WO-M or M-WO-P	50, 100, or 150 mg (titrated to tolerated dose)	P: HR: 77 ± 12, SBP: 123 ± 14, DBP: 82 ± 8; M: HR: 57 ± 11, SBP: 120 ± 20, DBP: 75 ± 9		P: FC 1: 0, FC 2: 18, FC 3: 11; M: FC 1: 3, FC 2: 22, FC 3: 4	M: 1.1 ± 0.2, LVEF: P: 69 ± 9; M: 71 ± 8	P: rest: 72 (28-87), Valsalva: 119 (89-165), PeE: 62 (31-113), PoE: 115 (55-171); M: rest: 25 (15-58), Valsalva: 94 (40-144), PeE: 28 (18-40), PoE: 45 (24-100)
Gilligan et al. (1993) [20]	Three-period CO between P, N, and V	V: 240 mg OD or BD, N: 80 mg OD or BD (titrated to tolerated dose)	P: HR: 78 ± 18, SBP: 126 ± 17, DBP: 75 ± 14; N: HR: 56 ± 8, SBP: 119 ± 20, DBP: 70 ± 14; V: HR: 66 ± 10, SBP: 126 ± 10, DBP: 73 ± 12		N/A	N/A	N/A
Toshima et al. (1986) [21]	Seq A: D-WO-V, Seq B: V-WO-D	D: 180 mg OD, V: 240 mg OD	Seq A: D: HR: 61 ± 9, SBP: 113 ± 15, DBP: 60 ± 8; V: HR: 62 ± 7, SBP: 115 ± 12, DBP: 67 ± 8	Seq B: V: HR: 60 ± 11, SBP: 120 ± 15, DBP: 69 ± 12; D: HR: 61 ± 11, SBP: 116 ± 11, DBP: 66 ± 12	N/A	LVEF: Seq A: D: 78 ± 7, V: 79 ± 8; Seq B: V: 85 ± 8, D: 84 ± 13	N/A
Bayonas-Ruiz et al. (2022) [23]	V: Bonow et al., Lösse et al.	N/A	N/A		Lösse et al.: FC: 2.4 ± 0.5	Bonow et al.: LVEF: 71± 9	N/A

Discussion

Dybro et al. [19] demonstrated in their trials that the LVOT gradients were significantly lower at rest, at peak exercise, and five minutes post-exercise during metoprolol treatment than during placebo treatment. The LVOT gradients during metoprolol were 25 mm Hg (IQR: 15-58 mm Hg) versus 72 mm Hg (IQR: 28-87 mm Hg) (P = 0.007) at rest, 28 mm Hg (IQR: 18-40 mm Hg) versus 62 mm Hg (IQR: 31-113 mm Hg) (P < 0.001) at peak exercise, and 45 mm Hg (IQR: 24-100 mm Hg) versus 115 mm Hg (IQR: 55-171 mm Hg) (P < 0.001) post-exercise [19]. The LVOT gradient during the Valsalva maneuver showed a similar trend during metoprolol treatment: 94 mm Hg (IQR: 40-144 mm Hg) versus 119 mm Hg (IQR: 89-165 mm Hg) (P = 0.08) [19]. The resting LVOT gradient was lower during metoprolol in 22 (76%) patients. In 21 (72%) patients, this difference was ≥20% compared with a placebo. The average difference in LVOT gradient during metoprolol treatment was 33 ± 25 mm Hg at rest, 66 ± 48 mm Hg at Valsalva maneuver, 47 ± 36 mm Hg at peak exercise, and 71 ± 63 mm Hg post-exercise [19]. They also demonstrated that metoprolol provided symptomatic relief to patients regarding NYHA class. The number of patients in NYHA functional class III or higher was 38% during placebo treatment versus 14% during metoprolol treatment (P < 0.01) [19]. The resting heart rate was 25% lower during metoprolol (57 ± 11 beats/minute versus 77 ± 12 beats/minute) (P < 0.0001). Heart rate at peak exercise was also lower with metoprolol treatment (107 ± 19 beats/minute versus 138 ± 23 beats/minute) (P < 0.001) [19]. However, metoprolol treatment did not affect LVEF (71% ± 8% versus 69% ± 9%) (P = 0.09), and invasive hemodynamic measures obtained at rest showed slightly elevated mean right atrial pressure, mean pulmonary pressure, and pulmonary capillary wedge pressure, which did not differ between treatments [19].

Gilligan et al. [20] tested verapamil against a beta-blocker called nadolol. They found that both drugs reduced heart rate at rest, during daily activities, and during peak exercise and that the beta-blocker was more effective than verapamil. They also measured exercise capacity in patients, which was not summarized in the table but is helpful to be mentioned as a part of the discussion. Exercise capacity was measured by measuring their exercise duration (in seconds), their oxygen consumption (in mL/kg/minute), and their anaerobic threshold (in mL/kg/minute), and it was found that nadolol reduced the peak exercise workload in 80% of patients. In contrast, verapamil had a more variable effect, which benefited verapamil [20].

Toshima et al. [21] compared verapamil to another calcium channel blocker belonging to the same class, diltiazem. There are two sequences in which drugs were given. Sequence A was given diltiazem first, followed by a washout period of one week, and then verapamil. Sequence B was the opposite. Firstly, they found that in sequence B, verapamil decreased heart rate significantly; the same was not seen in sequence A [21]. There were no significant changes in blood pressure in either sequence. Verapamil increased exercise duration significantly from 12.8 ± 3.8 to 14.8 ± 4.2 minutes in the first treatment period and from 15.2 ± 4.0 to 17.5 ± 4.5 minutes in the second period [21]. Maximal oxygen consumption was significantly augmented with verapamil from 22.6 ± 3.9 to 25.5 ± 6.1 mL/kg/minute in the first treatment period and from 25.7 ± 6.5 to 29.5 ± 7.3 mL/kg/minute in the second treatment period [21].

Bayonas-Ruiz et al. [23], in a systematic review that was also a meta-analysis, looked at all treatments for HCM. This included a pharmacological approach involving all drug classes related to the treatment of HCM and the invasive management of HCM. For this review, we only selected the results and conclusions they had reached from studying the drugs related to this review, which meant extracting data from the following studies: Bratt et al. (metoprolol), Bonow et al. (verapamil), Hanrath et al. (verapamil), and Lösse et al. (verapamil) [23]. Lösse et al. demonstrated that verapamil therapy improved the symptoms and quality of life of patients through the NYHA. The mean NYHA functional class of patients before initiating therapy was 2.8 ± 0.6, which was reduced to 2.4 ± 0.5 after treatment, which was statistically significant [23]. However, they could not conclude the pharmacological effect on the LVOT gradient and found that CCB produced no significant difference in LVEF.

Side Effect Profile of Metoprolol and Verapamil

Both drugs are commonly used to treat symptomatic HCM and are regularly prescribed. However, it is essential to discuss their potential side effects and what these studies have found regarding the use of these drugs. In the crossover trials covered in this review, 50 patients were receiving verapamil therapy. The results showed that eight patients had suffered from constipation, one patient developed atrioventricular (AV) dissociation, one more developed dizziness, and one more developed a headache. However, these were all minor, and it did not require them to stop the medication for this reason. However, three patients did discontinue verapamil therapy. One patient developed sinus arrest with a junctional escape beat, which disappeared spontaneously two hours after it had developed. There was no evidence suggesting sick sinus syndrome before the initiation of the trial [20,21]. Two more patients were required to discontinue verapamil due to severe fatigue and dizziness, at which point they could no longer tolerate the drug. During verapamil treatment, no abnormalities were developed in blood chemistry, hematology, or urine analysis. Twenty-nine patients received metoprolol therapy, and there were no serious adverse events related to the study drug. Three patients reported dizziness during treatment, and the dosage was reduced to 50 mg (one patient) and 100 mg (two patients). One patient experienced fatigue during both treatment periods (placebo and intervention), and metoprolol was reduced to 100 mg. Some transient side effects related to metoprolol treatment were reported, none of which prompted a reduction of dosage: two patients reported a cold sensation in the hands and feet, two patients reported diarrhea, one patient reported unusual tiredness, and one patient reported tingling in the hands or feet. No abnormalities of heart rhythm or electrical conduction on electrocardiograms were found [19].

Limitations

This study has its limitations. Significant heterogeneity exists due to the differences in study design, the population across the trials, drug doses, inclusion criteria, and clinical outcomes measured. The reports on the reduction of LVOTO are limited and are not measured uniformly across each study. Some studies did not mention the presence of the LVOT gradient either at rest or after exercise; they did not provide data for populations of obstructive and non-obstructive patients. Some studies measured the patient's quality of life and symptoms using the NYHA functional class, while others did not. This study should have addressed the question of the efficacy of long-term drug therapy, as the duration of the studies selected was short-term. However, the studies included for review did not mention anything on the topic. Another limitation is that this study only used studies that were available as free full texts; restricting studies that were not freely available may introduce bias into the results. It remains unclear whether the effect of metoprolol or verapamil provides a long-term reduction of LVOT gradients and symptoms or whether the effect subsides with long-term treatment. More studies should be done to compare the two drugs against one another.

## Conclusions

Hypertrophic cardiomyopathy (HCM) is a common cardiac disease that progresses quickly. It has severe complications that negatively impact a patient's quality of life and can cause physical and emotional stress. The technological advancement and the continuous development in our understanding of such a complex disease allow us to take steps that help improve its prognosis. Both drugs have shown potential for treating patients with symptomatic HCM. In the studies selected that focused on metoprolol, it proved effective in managing the symptoms of HCM. It showed a reduction in LVOT obstruction and an improvement in symptoms and quality of life in patients through an improvement in their NYHA functional class. Verapamil has also shown the ability to improve functional class; however, from the studies drawn, it did not show any reduction in LVOT or LVEF. Both drugs can be tailored for their use depending on the patient’s presentation. Patients who present with severe LVOT obstruction may benefit from metoprolol, whereas patients who require a more variable effect in managing their baseline hemodynamics and require an improvement in functional class may benefit from verapamil. Both drugs have a good side effect profile and have been proven safe to use in the treatment of HCM. However, more studies with longer follow-up periods are required to establish if either drug provides a long-term reduction of LVOT gradients, whether symptoms would subside with long treatment, or if the drug loses efficacy as treatment progresses. Metoprolol has been shown to reduce the LVOT gradient in the short term, and verapamil has been shown to improve exercise capacity, but these effects need to be shown to be stable over time to get a clear picture of the best way to treat HCM with drugs. It would also be suitable to conduct studies that directly compare the two using crossover trials, similar to those used in the studies that were included in this article.

## References

[1] Maron BJ (2002). Hypertrophic cardiomyopathy: a systematic review. JAMA.

[2] Baudhuin LM, Kotzer KE, Kluge ML, Maleszewski JJ (2015). What is the true prevalence of hypertrophic cardiomyopathy?. J Am Coll Cardiol.

[3] Marian AJ, Braunwald E (2017). Hypertrophic cardiomyopathy: genetics, pathogenesis, clinical manifestations, diagnosis, and therapy. Circ Res.

[4] (2023). MedlinePlus: Familial hypertrophic cardiomyopathy. https://medlineplus.gov/genetics/condition/familial-hypertrophic-cardiomyopathy/.

[5] Riddle DL, Blumenthal T, Meyer BJ (1997). Section II: the organization, structure, and function of muscle. C. elegans II, 2nd edition.

[6] (2023). American Heart Association, Inc.: Hypertrophic cardiomyopathy (HCM). https://www.heart.org/en/health-topics/cardiomyopathy/what-is-cardiomyopathy-in-adults/hypertrophic-cardiomyopathy.

[7] Wigle ED (2001). Cardiomyopathy: the diagnosis of hypertrophic cardiomyopathy. Heart.

[8] Sakamoto T, Tei C, Murayama M, Ichiyasu H, Hada Y (1976). Giant T wave inversion as a manifestation of asymmetrical apical hypertrophy (AAH) of the left ventricle. Echocardiographic and ultrasono-cardiotomographic study. Jpn Heart J.

[9] Grigg LE, Wigle ED, Williams WG, Daniel LB, Rakowski H (1992). Transesophageal Doppler echocardiography in obstructive hypertrophic cardiomyopathy: clarification of pathophysiology and importance in intraoperative decision making. J Am Coll Cardiol.

[10] Yu EH, Omran AS, Wigle ED, Williams WG, Siu SC, Rakowski H (2000). Mitral regurgitation in hypertrophic obstructive cardiomyopathy: relationship to obstruction and relief with myectomy. J Am Coll Cardiol.

[11] Kizilbash AM, Heinle SK, Grayburn PA (1998). Spontaneous variability of left ventricular outflow tract gradient in hypertrophic obstructive cardiomyopathy. Circulation.

[12] Slama M, Tribouilloy C, Maizel J (2016). Left ventricular outflow tract obstruction in ICU patients. Curr Opin Crit Care.

[13] Kusunose K, Zheng R, Yamada H, Sata M (2022). How to standardize the measurement of left ventricular ejection fraction. J Med Ultrason (2001).

[14] Suboc T (2023). MSD Manual Professional Edition: Hypertrophic cardiomyopathy. https://www.msdmanuals.com/professional/cardiovascular-disorders/cardiomyopathies/hypertrophic-cardiomyopathy.

[15] Morris J, Dunham A (2022). Metoprolol. https://www.ncbi.nlm.nih.gov/books/NBK532923/.

[16] Fahie S, Cassagnol M (2023). Verapamil. https://www.ncbi.nlm.nih.gov/books/NBK538495/.

[17] Page MJ, McKenzie JE, Bossuyt PM (2021). The PRISMA 2020 statement: an updated guideline for reporting systematic reviews. BMJ.

[18] Sterne JA, Savović J, Page MJ (2019). RoB 2: a revised tool for assessing risk of bias in randomised trials. BMJ.

[19] Dybro AM, Rasmussen TB, Nielsen RR, Andersen MJ, Jensen MK, Poulsen SH (2021). Randomized trial of metoprolol in patients with obstructive hypertrophic cardiomyopathy. J Am Coll Cardiol.

[20] Gilligan DM, Chan WL, Joshi J, Clarke P, Fletcher A, Krikler S, Oakley CM (1993). A double-blind, placebo-controlled crossover trial of nadolol and verapamil in mild and moderately symptomatic hypertrophic cardiomyopathy. J Am Coll Cardiol.

[21] Toshima H, Koga Y, Nagata H, Toyomasu K, Itaya K, Matoba T (1986). Comparable effects of oral diltiazem and verapamil in the treatment of hypertrophic cardiomyopathy. Double-blind crossover study. Jpn Heart J.

[22] Shea BJ, Reeves BC, Wells G (2017). AMSTAR 2: a critical appraisal tool for systematic reviews that include randomised or non-randomised studies of healthcare interventions, or both. BMJ.

[23] Bayonas-Ruiz A, Muñoz-Franco FM, Sabater-Molina M, Oliva-Sandoval MJ, Gimeno JR, Bonacasa B (2023). Current therapies for hypertrophic cardiomyopathy: a systematic review and meta-analysis of the literature. ESC Heart Fail.

